# Follicular lymphoma without lymphadenopathy incidentally diagnosed by sentinel lymph node biopsy during breast cancer surgery: a case report

**DOI:** 10.1186/s40792-022-01524-4

**Published:** 2022-09-13

**Authors:** Emiko Hiraoka, Norio Masumoto, Takaoki Furukawa, Norimasa Kuraoka, Ichiro Nagamine, Aya Kido, Kazuhiro Sentani, Sunao Ootagaki

**Affiliations:** 1 Department of Surgery, Hiroshima Kyoritsu Hospital, 2-20-20 Nakasu Asaminami-Ku, Hiroshima, Hiroshima 731-0121 Japan; 2 Department of Breast Surgery, Hiroshima University Hospital, 1-2-3 Kasumi, Minami-Ku, Hiroshima, Hiroshima 734-8551 Japan; 3 Graduate School of Biomedical and Health Sciences, Hiroshima University, 1-2-3 Kasumi, Minami-Ku, Hiroshima, Hiroshima 734-8551 Japan

**Keywords:** Breast cancer, Malignancy, Follicular lymphoma, Sentinel lymph node biopsy

## Abstract

**Background:**

Concurrent breast cancer and malignant lymphoma is a rare phenomenon. This report describes malignant lymphoma that was incidentally diagnosed from a sentinel lymph node biopsy (SLNB) during breast cancer surgery.

**Case presentation:**

A 73-year-old woman with a history of ovarian cancer and diabetes presented with right focal asymmetric density on a mammogram acquired during routine breast cancer screening. Ultrasonography (US) and magnetic resonance imaging (MRI) showed a 13.5-mm tumor in the upper lateral region of the right breast. A US-guided Mammotome biopsy revealed invasive ductal carcinoma of the right breast. Preoperative assessments including positron emission tomography–computerized tomography, found no evidence of axillary lymphadenopathy or distant metastasis. Because the breast cancer was stage I, the patient underwent a right mastectomy and a sentinel lymph node biopsy (SLNB) at our hospital. Pathological assessment of the biopsy revealed follicular lymphoma (FL), but no metastatic breast cancer. The patient was referred to hematology to stage the FL. Bone marrow findings were negative and stage I FL was diagnosed. After the mastectomy, she was monitored and given adjuvant therapy with an aromatase inhibitor.

**Conclusions:**

Follicular lymphoma was incidentally diagnosed from an SLNB obtained to determine the dissemination of early-stage breast cancer. Collaboration with hematologists is important to determine optimal treatment plans for such patients regardless of the rarity of such events.

## Background

Chemotherapy or radiation therapy for malignant lymphoma (ML) increases the risk of developing secondary breast cancer [1]. On the other hand, ML and breast cancer are rarely diagnosed simultaneously [2]. Most previous reports of synchronous breast cancer and ML describe lymphadenopathy in the axilla or other sites during preoperative assessment for breast cancer [2]. Sentinel lymph node biopsies (SLNBs) are usually obtained when early-stage breast cancer is not accompanied by obvious lymph node metastasis. Thus, a diagnosis of ML by SLNB during breast cancer surgery is extremely rare [3]. This report describes follicular lymphoma (FL) diagnosed by SLNB for stage I breast cancer.

## Case presentation

**Fig. 1:**
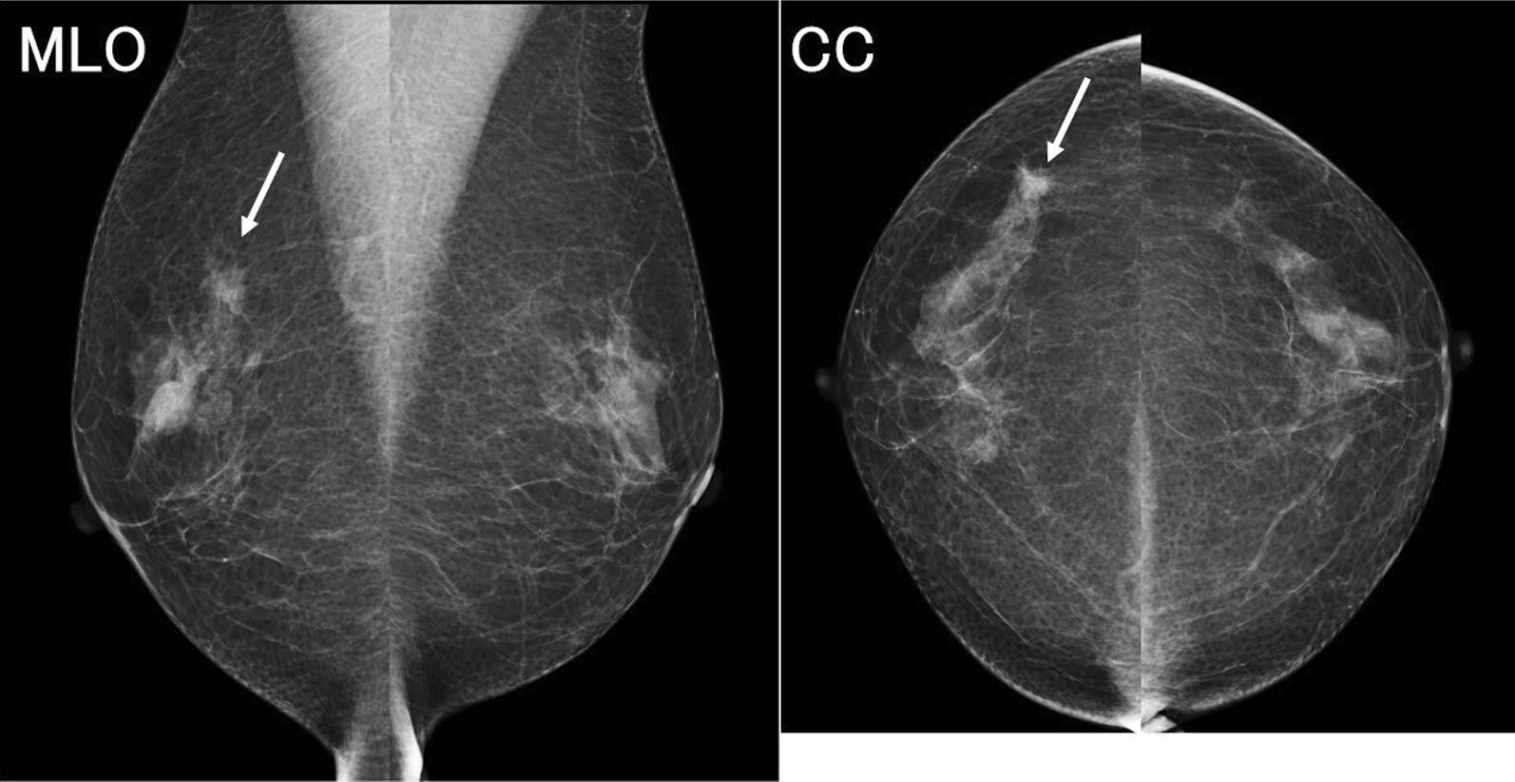
Mammogram findings. Right mediolateral oblique (MLO) and craniocaudal (CC) views of mammograms show focal asymmetric density (arrows)

**Fig. 2:**
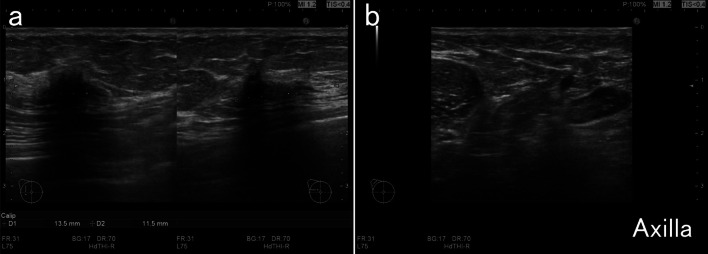
Ultrasound findings of breast. **a** Irregular hypoechoic mass (13.5 mm) in right upper lateral zone. **b** Lymphadenopathy is not obvious in right axilla

**Fig. 3:**
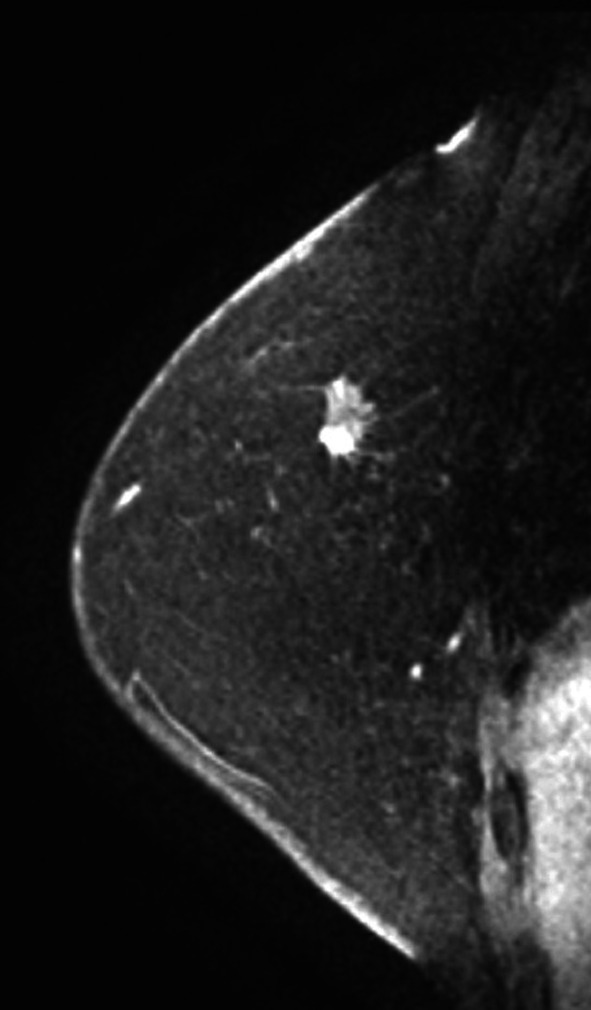
Contrast-enhanced MRI findings of breast. Spiculated, contrast-enhanced, 12-mm mass in right upper lateral zone. Lymphadenopathy is not obvious in right axilla. MRI, magnetic resonance imaging

**Fig. 4:**
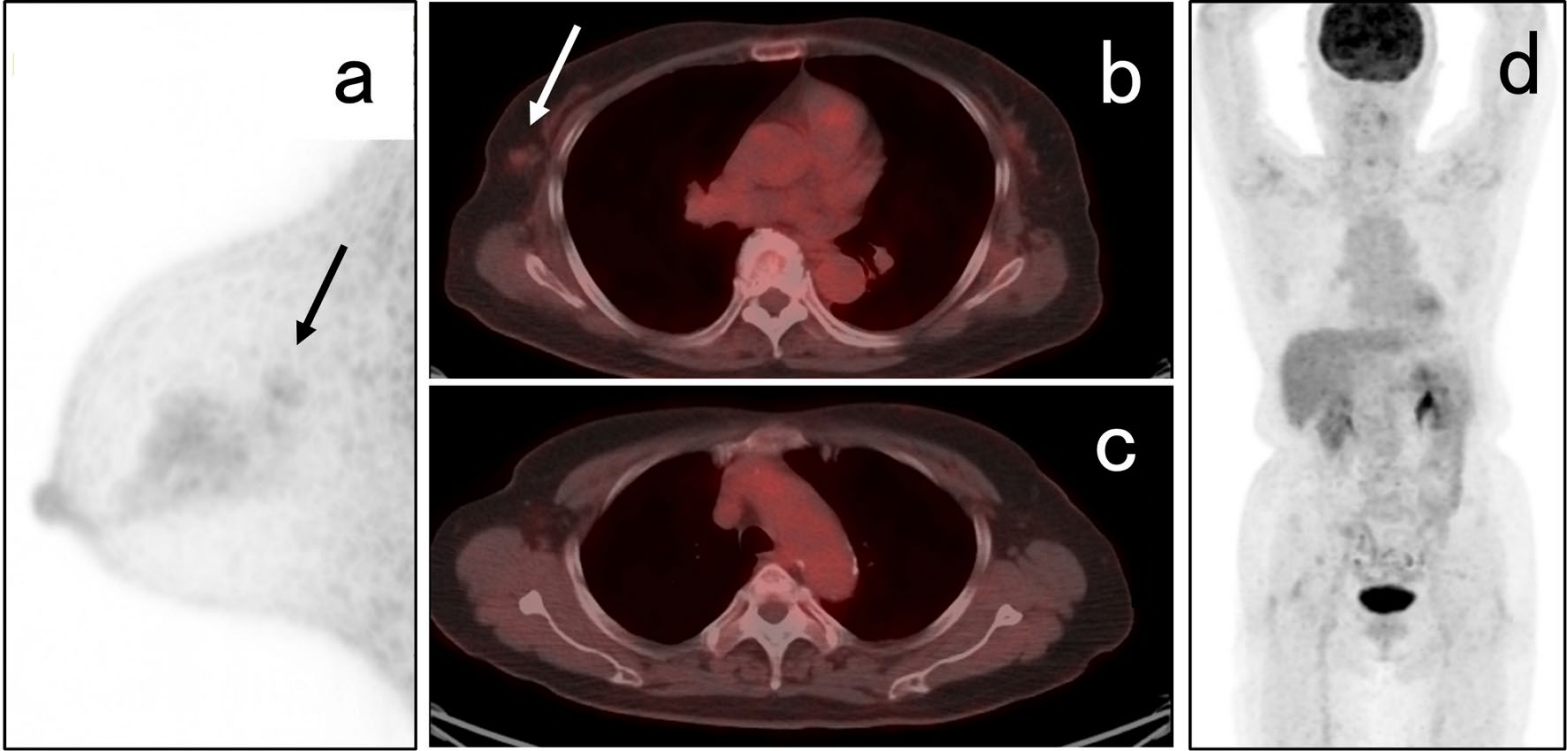
Findings of db-PET and whole-body PET–(CT). **a**, **b** Uptake with SUVmax 2.1 and 1.4 (arrows) in right breast. **c**, **d** Uptake is not significant in right axilla and organs. *CT* computerized tomography, *dbPET* dedicated breast positron emission tomography, *SUVmax* maximum standard uptake value

A 73-year-old woman presented with right focal asymmetric density determined by mammography during a breast cancer screening in December 2021 (Fig. 1). Her medical history included ovarian cancer at the age of 48 years and diabetes with HbA1c 7.1% under treatment. She had undergone a simple hysterectomy, bilateral salpingo-oophorectomies, pelvic lymph node dissection, partial omentectomy, and appendicectomy for stage C ovarian cancer at another hospital 25 years previously. Details of the histological type of ovarian cancer determined at that time were not available. She had been treated postoperatively with 5 cycles of cyclophosphamide and cisplatin and remained free of recurrence for the next 10 years. Therefore, follow-up was terminated. Her family history was negative for cancer. Palpation and visual examination revealed no obvious mass in the right breast or right axillary lymphadenopathy. Ultrasonography (US) revealed an upper lateral, 13.5-mm, irregular hypoechoic mass in the right breast (Fig. 2a), but no obvious lymphadenopathy in the right axilla (Fig. 2b). The mass was diagnosed as invasive ductal carcinoma of the breast by a US-guided Mammotome biopsy. Contrast-enhanced magnetic resonance imaging (MRI) of the breast revealed an upper lateral, 12-mm, contrast-enhanced mass with spiculation in the right breast, but no obvious lymph node enlargement (Fig. 3). Dedicated breast positron emission tomography (PET) showed significant accumulation with a maximum standardized uptake value (SUVmax) of 2.1 in the right breast (Fig. 4a). Whole-body PET–computerized tomography (CT) showed significant uptake (SUVmax 1.4) in the right breast (Fig. 4b), but none in the right axilla to suggest lymph node metastasis (Fig. 4c), and distant metastasis was not suggested (Fig. 4d). Right upper lateral breast cancer cT1cN0M0 stage I was diagnosed based on these findings. The patient underwent right mastectomy in January 2022, when we obtained an SLNB specimen. An intraoperative rapid pathological assessment of the node by indigo carmine staining was negative for breast cancer metastasis. The postoperative course was uneventful and she was discharged on postoperative day 6.

**Fig. 5:**
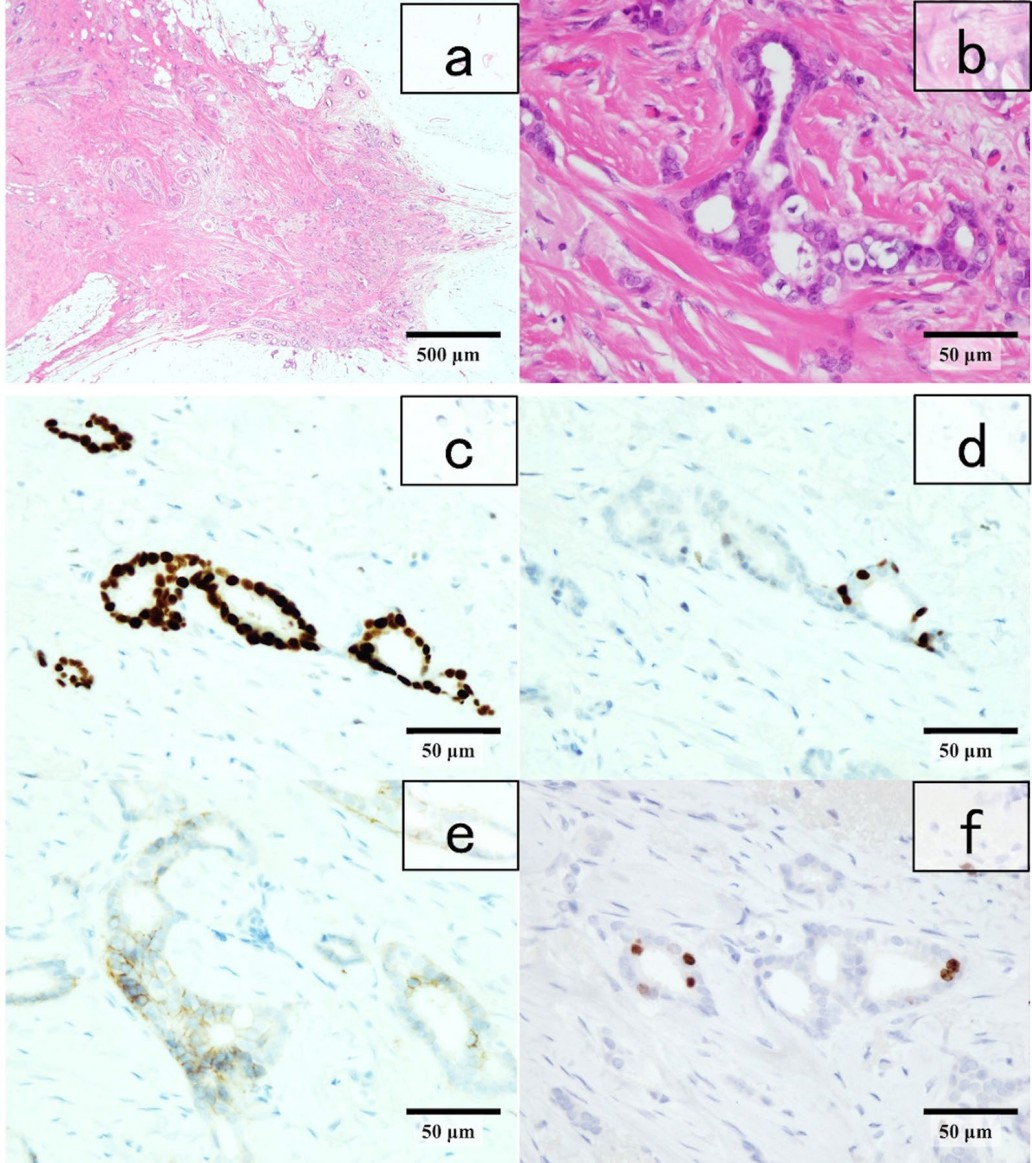
Postoperative histopathological findings of breast tumor. **a**, **b** Invasive ductal carcinoma, tubule forming type, nuclear grade 1, histological grade I (hematoxylin–eosin stain; magnification, × 40, × 400). Immunostaining findings: **c** ER ( +), **d** PR ( +), **e** HER2 score 2, **f** Ki-67 score 5% in right breast (× 400), and HER2 amplification is absent in FISH. *ER* estrogen receptor, *FISH* fluorescent in situ hybridization, *HER2* human epidermal growth factor receptor-2, *PR* progesterone receptor

**Fig. 6:**
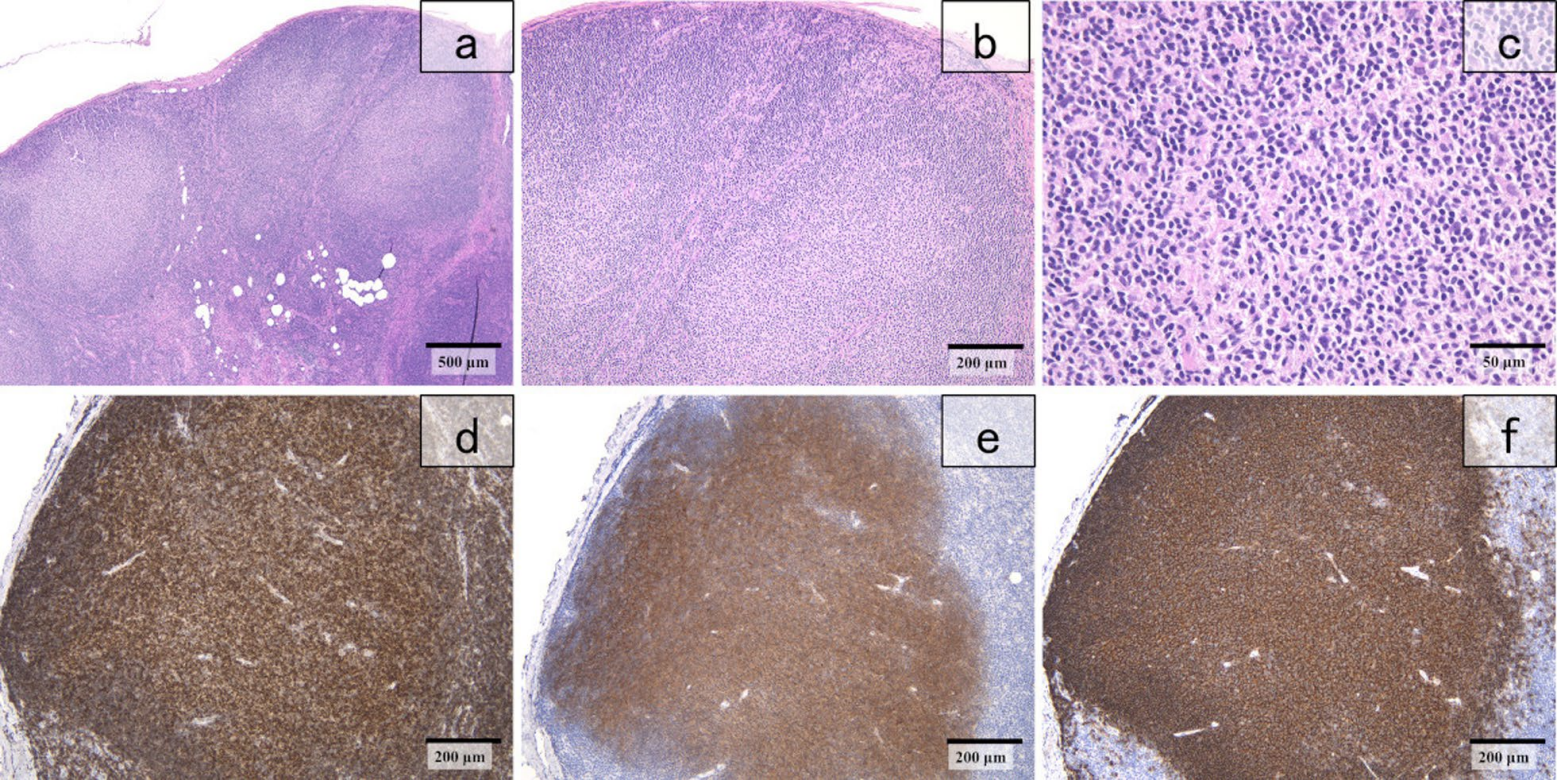
Histopathological findings of sentinel lymph node. **a**–**c** Nodular proliferation of follicular lymphoma (hematoxylin–eosin stain; magnification × 4, × 100, × 400). Immunostained lymphoma cells are **d** CD20^+^, **e** CD10^+^, and **f** BCL-2^+^. Magnification, × 100

The postoperative pathology results of the right breast revealed invasive ductal carcinoma, tubule forming type, pT1c (11 mm), Ly0, V0, f, nuclear grade 1, histological grade 1 (Fig. 5a and b), estrogen receptor (ER) J-score 3b (Fig. 5c) and progesterone receptor (PgR) J-score 2 (Fig. 5d), human epidermal growth factor receptor-2 (HER2) score 2 (Fig. 5e), no amplification of HER2 according to fluorescent in situ hybridization (FISH), and Ki-67 score, 5% (Fig. 5f). Breast cancer metastasis was negative in the sentinel lymph node. On the other hand, proliferative cells slightly larger than lymphocytes were evident in a nodular structure within this node (Fig. 6a–c), but tingible body macrophages (TBMs) were not evident. Immunostaining revealed diffusely expressed tumor cells CD20 (Fig. 6d) and surrounding small CD3^+^ and CD5^+^ lymphocytes. The cells in germinal centers were CD10^+^ and Bcl-2^+^ (Fig. 6e and f, respectively). These findings indicated a diagnosis of Grade 1–2 FL.

Since the breast cancer was pT1cN0M0 Stage IA, we considered that the prognosis of our patient would be determined according to the stage of FL and referred her to a hematologist. Her bone marrow results were negative and stage I FL was confirmed. The treatment plan was observation, and she was prescribed with an aromatase inhibitor as postoperative endocrine therapy for breast cancer. She will remain on aromatase inhibitors for at least 5 years and will be followed up for 10 years after the breast cancer surgery.

## Discussion

Sentinel lymph node biopsies during surgery for early breast cancer have become an established surgical procedure [4] with which to assess metastatic breast cancer in axillary lymph nodes. This is important because axillary lymph nodes do not require dissection when sentinel lymph nodes are negative. Patients with breast cancer who undergo SLNB usually have no lymphadenopathy on preoperative images and/or no lymph node metastasis determined by preoperative cytology or biopsy. Malignant lymphoma is rarely considered when patients with breast cancer have no obvious lymphadenopathy in axillae or elsewhere. Therefore, only three publications describe ML diagnosed from SLNB obtained to determine metastatic breast cancer [2, 3, 5].

Thus, our patient is only the fourth with ML diagnosed in this manner according to the English literature. One of the previous patients had mantle cell lymphoma (MCL) and the remainder (including our patient) had FL (Table 1). Non-Hodgkin lymphoma (NHL) was the predominant type of ML, and both MCL and FL are classified as NHL [6]. Woo et al. found MCL and FL in 3 (7.8%) and 10 (26.3%) of 38 patients, respectively, with synchronous breast cancer and NHL [2]. Meanwhile, the general incidences of MCL and FL [7] in NHL are 4.1% and 17%, respectively. The final breast cancer stages of the four patients were all IA, but the lymphoma stages differed. The three patients with NHL diagnosed by SLNB were postoperatively staged by PET–CT, which identified one each with advanced stages III and IV lymphoma [2, 3, 5]. Our patient was preoperatively assessed by PET–CT for ovarian cancer, which was in her medical history. Although PET–CT is useful for staging ML [8], lymphadenopathy was not obvious in preoperative PET–CT images of our patient, whereas FL was diagnosed from an SLNB to determine metastatic breast cancer, which is truly rare. Most ML detected at the time of surgery for breast cancer has been found in non-sentinel nodes [3]. Axillary dissection can be omitted if a sentinel node biopsy is negative in patients aged ≥ 70 years who are more susceptible to ML [6]. However, ML in non-sentinel nodes might be overlooked. The NCCN guidelines [9] do not indicate whole-body CT or PET–CT for early-stage breast cancer without obvious clinical symptoms or axillary lymphadenopathy. Thus, early detection of patients with asymptomatic ML in non-sentinel nodes may be difficult.

**Table 1:** Malignant lymphomas in women diagnosed by sentinel lymph node biopsies during breast cancer surgery

Characteristics	Report no.			
	1	2	3	4
Age (y)	74	67	60	73
Breast cancer indicators	pT1cN0M0	pT1cN0M0	pT1bN0M0	pT1cN0M0
Stage	IA	IA	IA	IA
Surgery	Bp + SNLB	Bp + SNLB	Bp + SNLB	Bt + SNLB
Histology	ILC	IDC	IDC	IDC
Breast cancer subtype	ER + /PgR + /HER2−	ER−/PgR−/HER2 +	ER−/PgR−/HER2 +	ER + /PgR + /HER2−
Adjuvant therapy for breast cancer	Radiotherapy followed by AI	Trastuzumab with R-DHAP	Trastuzumab	AI
Lymphadenopathy in preoperative images	None	Axillary lymph node	None	None
Lymphoma stage	I	IV	III	I
Subtype	FL	MCL	FL	FL
Therapy	None	R-CHOP then R-DHAP	R-CHOP	None
Priority for PT	Breast cancer	MCL	FL	Breast cancer
Year published	2010	2016	2019	2022
References	[5]	[2]	[3]	Present study

*AI* aromatase inhibitor, *Bp* breast partial mastectomy, *Bt* breast total mastectomy, *FL* follicular lymphoma, *HER2* human epidermal growth factor receptor type2, *IDC* invasive ductal carcinoma, *ILC* invasive lobular carcinoma, *MCL* mantle cell lymphoma, *PT* postoperative treatment, *R-CHOP* rituximab, cyclophosphamide, doxorubicin hydrochloride, oncovin, and prednisolone, *R-DHAP* rituximab, dexamethasone, high-dose ara-C, cytarabine, and cisplatin, *SNLB* sentinel lymph node biopsy, *TNBC* triple-negative breast cancer

The question arises as to which treatment should be administered first to patients with concurrent breast cancer and ML. The progression of breast cancer and ML has been compared and prioritized according to a requirement for chemotherapy and radiation therapy [2, 3, 5]. The 5- and 10-year survival rates of low-grade NHL are 82% and 73%, respectively, even when followed up at an advanced stage and without treatment [10]. A retrospective study of stage I FL found equally good progression-free survival (PFS) between patients who were not treated or treated only with chemotherapy and those who received radiation therapy [11]. The treatment plan for our patient was follow-up for stage I low-grade FL and postoperative endocrine therapy for breast cancer immediately after FL was staged.

The next question is whether SLNB will be false negative if ML is found in sentinel nodes. One report indicated a false-negative SLNB from a patient with Waldenström macroglobulinemia, which is an indolent type of lymphoma. This was because the sentinel lymph node had no breast cancer metastasis, but contained proliferating lymphoma cells, whereas a non-sentinel lymph node had metastatic breast cancer [12]. Both negative and positive lymphomas have been accurately diagnosed from SLNBs in nine patients with breast cancer and a history of lymphoproliferative disease [3]. Compared with lymphomas arising from the lymph medulla such as Waldenström macroglobulinemia, those such as FL that arise from the lymph cortex are generally considered not to inhibit lymph flow [14]. Some types of lymphoma can be identified in sentinel lymph nodes that do not obstruct lymphatic flow, as they interfere with the ability of the dye and tracer used in SLNB to pass through. However, when breast cancer cells have difficulty growing in lymph nodes due to concomitant lymphoma, an SLNB might lose its original significance as the initial site of breast cancer metastasis. Non-sentinel nodes should also be sampled when coexisting breast cancer and lymphoma are preoperatively confirmed, or if lymphoma is diagnosed by rapid SLNB, considering the possibility of false-negative sentinel nodes. Barranger et al. also described axillary dissection due to positive metastasis in SLNBs, and all 21 lymph nodes sampled were positive for follicular lymphoma [15]. Postoperative follow-up requires thorough axillary observation by palpation and US, due to the possibility of false-negative SLNB results and residual FL in non-sentinel nodes.

## Conclusions

We described a patient with very rare FL that was incidentally detected in an SLNB obtained to assess metastatic breast cancer. Cooperation with a hematologist is important to optimize treatment for such patients.

## Data Availability

All data generated or analyzed in this study are included in this report.

## Abbreviations

CT: Computerized tomography
FL: Follicular lymphoma
ML: Malignant lymphoma
MRI: Magnetic resonance imaging
NHL: Non-Hodgkin lymphoma
PET: Positron emission tomography
SLNB: Sentinel lymph node biopsy
US: Ultrasonography

## References

[1] Lewis WD, Lilly S, Jones KL (2020). Lymphoma: diagnosis and treatment. Am Fam Physician.

[2] Woo EJ, Baugh AD, Ching K (2016). Synchronous presentation of invasive ductal carcinoma and mantle cell lymphoma: a diagnostic challenge in menopausal patients. J Surg Case Rep.

[3] Fushimi A, Kinoshita S, Kudo R, Takeyama H (2019). Incidental discovery of follicular lymphoma by sentinel lymph node biopsy and skin-sparing mastectomy for Paget's disease associated with invasive breast cancer. J Surg Case Rep.

[4] Lyman GH, Somerfield MR, Giuliano AE (2017). Sentinel lymph node biopsy for patients with early-stage breast cancer: 2016 American Society of Clinical Oncology Clinical Practice Guideline Update Summary. J Oncol Pract.

[5] Cuff KE, Dettrick AJ, Chern B (2010). Synchronous breast cancer and lymphoma: a case series and a review of the literature. J Clin Pathol.

[6] Jaffe ES. Introduction and overview of the classification of lymphoid neoplasms. In: Swerdlow SH, et al. editors. WHO classification of tumours of haematopoietic and lymphoid tissues WHO classification of tumours, revised 4th edition, Volume 2. Lyon, IARC; 2017: pp. 266–279.

[7] Al-Hamadani M, Habermann TM, Cerhan JR, Macon WR, Maurer MJ, Go RS (2015). Non-Hodgkin lymphoma subtype distribution, geodemographic patterns, and survival in the US: a longitudinal analysis of the National Cancer Data Base from 1998 to 2011. Am J Hematol.

[8] Jiang H, Li A, Ji Z, Tian M, Zhang H (2022). Role of radiomics-based baseline PET/CT imaging in lymphoma: diagnosis, prognosis, and response assessment. Mol Imaging Biol.

[9] Gradishar WJ (2020). Breast Cancer, Version 3.2020, NCCN Clinical Practice Guidelines in Oncology. J Natl Compr Canc Netw.

[10] Horning SJ, Rosenberg SA (1984). The natural history of initially untreated low-grade non-Hodgkin's lymphomas. N Engl J Med.

[11] Friedberg JW, Byrtek M, Link BK, Flowers C, Taylor M, Hainsworth J, Cerhan JR, Zelenetz AD, Hirata J, Miller TP (2012). Effectiveness of first-line management strategies for stage I follicular lymphoma: analysis of the National LymphoCare Study. J Clin Oncol.

[12] Benoit L, Arnould L, Collin F, Fraisse J, Cuisenier J, Chauffert B (2004). Concurrent lymphoma and metastatic breast carcinoma in the axillary, confounding sentinel lymph-node biopsy. Eur J Surg Oncol.

[13] Dy BM, Reynolds CA, Wahner-Roedler DL, Boughey JC (2010). Sentinel lymph node surgery for the staging of breast carcinoma in patients with lymphoproliferative disease. Am Surg.

[14] Arana S, Vasquez-Del-Aguila J, Espinosa-Bravo M, Peg V, Rubio IT (2013). Lymphatic mapping could not be impaired in the presence of breast carcinoma and coexisting small lymphocytic lymphoma. Am J Case Rep.

[15] Barranger E, Marpeau O, Uzan S, Antoine M (2005). Axillary sentinel node involvement by breast cancer coexisting with B-cell follicular lymphoma in non-sentinel nodes. Breast J.

